# High-Frequency (30 MHz–6 GHz) Breast Tissue Characterization Stabilized by Suction Force for Intraoperative Tumor Margin Assessment

**DOI:** 10.3390/diagnostics13020179

**Published:** 2023-01-04

**Authors:** Hadi Mokhtari Dowlatabad, Amir Mamdouh, Narges Yousefpour, Reihane Mahdavi, Ashkan Zandi, Parisa Hoseinpour, Seyed Mohammad Sadegh Moosavi-Kiasari, Fereshte Abbasvandi, Yasin Kordehlachin, Mohammad Parniani, Karim Mohammadpour-Aghdam, Pooya Faranoush, Mohammad Reza Foroughi-Gilvaee, Mohammad Abdolahad

**Affiliations:** 1 Nano Bioelectronics Devices Lab, Cancer Electronics Research Group, School of Electrical and Computer Engineering, Faculty of Engineering, University of Tehran, Tehran 14399-57131, Iran; 2 Department of Pathology, Breast Cancer Research Center, Motamed Cancer Institute, ACECR, Tehran 15179-64311, Iran; 3 ATMP Department, Breast Cancer Research Center, Motamed Cancer Institute, ACECR, Tehran 15179-64311, Iran; 4 Pathology Department, Breast Cancer Research Center, Motamed Cancer Institute, ACECR, Tehran 15179-64311, Iran; 5 Center of Excellence for Applied Electromagnetic Systems, University of Tehran, Tehran 14399-57131, Iran; 6 Pediatric Growth and Development Research Center, Institute of Endocrinology and Metabolism, Iran University of Medical Sciences, Tehran 14496-14535, Iran; 7 Cancer Electronics Research Center, Tehran University of Medical Sciences, Tehran 14197-33141, Iran

**Keywords:** dipolar polarization, GHz spectroscopy, scattering, breast cancer, tumor margin

## Abstract

A gigahertz (GHz) range antenna formed by a coaxial probe has been applied for sensing cancerous breast lesions in the scanning platform with the assistance of a suction tube. The sensor structure was a planar central layer and a metallic sheath of size of 3 cm^2^ connected to a network analyzer (keySight FieldFox N9918A) with operational bandwidth up to 26.5 GHz. Cancer tumor cells have significantly higher water content (as a dipolar molecule) than normal breast cells, changing their polarization responses and dielectric losses to incoming GHz-based stimulation. Principal component analysis named *S*_11_, related to the dispersion ratio of the input signal, is used as a parameter to identify malignant tumor cells in a mouse model (in vivo) and tumor specimens of breast cancer patients (in vitro) (both central and marginal parts). The results showed that *S*_11_ values in the frequency range from 5 to 6 GHz were significantly higher in cancer-involved breast lesions. Histopathological analysis was the gold standard for achieving the *S*_11_ calibration to distinguish normal from cancerous lesions. Our calibration on tumor specimens presented 82% positive predictive value (PPV), 100% negative predictive value (NPV), and 86% accuracy. Our goal is to apply this system as an in vivo non-invasive tumor margin scanner after further investigations in the future.

## 1. Introduction

The increased incidence of cancer is a subject of significant concern worldwide. Despite several technological advances in real-time presurgical and intraoperative cancer detection methods, there are still many discrepancies, with final permanent pathology results as the gold standard [1,2].

Intraoperative frozen pathology is the typical analysis for obtaining free tumor margins and decreasing the recurrence rate in cancer patients [3], but it does not meet all surgeons’ needs. Incomplete fixation of adipose cells existing in dissected breast tumor margins is a crucial pitfall in the accuracy of frozen pathology in breast cancer surgeries [4], so at least 20–30% misdiagnosis in breast tumor margins was reported in the literature [5,6]. Thus, several types of research have been conducted to fill the gap and invent precise intraoperative complementary devices [7,8]. One of the most attractive methods for cancer detection has been based on tissue dielectric properties [9]. Characteristics of this approach include real-time responses [10], precise diagnoses [11], compatibility with tissue pathophysiological status, and non-invasive detection techniques [12].

The response of biological tissues to high-frequency electromagnetic stimulation that leads to different polarization effects (electronic, atomic, dipolar, and interfacial) could be characterized by their conductivity (σ) and permittivity (ε) [13]. It strongly depends on the stimulation frequency due to different electrical currents passing through the tissue’s structure. Most of the electrical current at low frequencies passes through the extracellular matrix due to the α dispersion region, while it is capacitively coupled to the bilayer lipid cell membrane and intracellular elements in medium frequencies (β dispersion region) [14]. In the γ dispersion region (frequency > 1 GHz), all the current passes through the cell, the electrical response is strongly affected by the depolarization resonance of water molecules in the cells, and the dielectric properties of tissue at frequencies above 1 GHz reflect the dielectric relaxation of tissue water [15].

This research is focused on the characterization of breast tumors for margin evaluation purposes based on GHz spectroscopy of tissue. Due to the correlation between scattering parameters and dielectric properties, measuring scattering parameters is one of the ways to obtain dielectric properties [16]. The concept of scattering parameters is based on the transmitted power and reflected wave. *S*_11_ is an important parameter of our characterization, as the biological tissues are not one port transmission line [17]. It is known that the intracellular water content of cancer cells is much more than in normal cells [18]. Hence, *S*_11_, as the reflection wave parameter of GHz stimulation, may be different between normal and cancer tissues. Previous in vitro GHz detection systems on some human organs, such as the breast [19,20,21,22], colon [23,24], and liver [25,26] used standing probes, none of which were calibrated with permanent pathology. Furthermore, they were not applied for assessment of the intraoperative frozen section. Moreover, they used small sample sizes without functionalization and calibration of the technique.

The penetration depth of GHz fields into biological tissues is about 1 mm, which is compatible with the depth required to be investigated in the frozen-section pathology of margins [27]. Also, an important parameter affecting such observation is the quality of the contact surface between the GHz probe and the tissue [28].

Here, a GHz system based on a coaxial probe embedded in a customized Foley catheter and connected to a suction system was developed to evaluate the *S*_11_ difference between normal and cancer tissues in a mouse model and human in vitro breast specimens (*n* = 127). The main study was performed on breast tumor margins, but we used small samples from all types of breast tissue for GHz characterization and calibration. Suction was applied not only to stablize the probe/tissue contact interface but also to deplete the additive water of the tissue (such as non-intra-coastal water, which may perturb the GHz response). The reflection index of the wave recorded by the same stimulating probe was drastically affected with respect to its interaction with cancer cells due to their higher dipole resonance. A pathologically calibrated scoring was proposed as a cut-off value to distinguish between normal and malignant lesions with a feature size of about 50 mm^2^. This system may be helpful in real-time tumor margin scanning in surgical procedures.

## 2. Materials and Methods

### 2.1. Measurement and Data Processing

**Figure 1:**
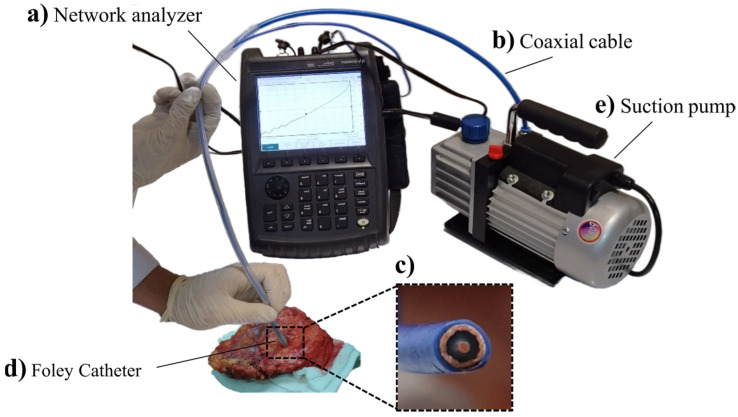
GHz measurement system. (**a**) The used network analyzer (KeySight FieldFox N9918A). (**b**) The open-ended coaxial cable (Semi-Flexible Cable 670-086 SXE). (**c**) Magnified view of the coaxial cable. (**d**) A Foley catheter modified for applying suction thoroughly. (**e**) Suction pump.

The network analyzer (GHz wave generation) and detection system (KeySight FieldFox N9918A) used in this study is a 0–26.5 GHz frequency spectroscopic actuator/detector (Figure 1a). It consists of a hand-held, flexible, open-ended coaxial cable (Semi-Flexible Cable 670-086 SXE) for actuating and sensing (Figure 1b,c). The coaxial cable was embedded in the center of a Foley catheter as a tube to apply the suction force (Figure 1d,e) to obtain the best physical contact between the probe (copper cladding) and tissue.

GHz electromagnetic waves are transmitted to the tissue through the coaxial probe’s tip at frequencies ranging from 0.1 to 6 GHz. These frequencies were selected based on the best distinctive water molecular dipole resonance response at high frequencies. The stimulation induces no destructive or ionizing damage to the tissues. The intensity of the wave transmitted across the probe’s opening is neglectable. The stimulation voltage of the probe is about 0.1 Volts which would not ionize the interacted tissue. The power reaching the tissue is less than 0.1 mW per square mm. The time interval of each signal transmission and response received due to the reflected signal is about 3 ms. The radius of the copper cladding end of the sensor cable is 0.4 mm.

### 2.2. Mouse and Model Test

The 4T1 cell line was injected subcutaneously into the mice models. After 10 days (when the tumors were formed as a massive specimen with about 5 to 10 mm diameter), the outer layer of the tumor was shaved, and the tumor with the fresh surface was chosen for GHz measurement in vivo. In this regard, the coaxial probe was connected to the tumor surface by applying suction force. Five subsequential measurements were carried out on each tumor. Moreover, a normal superficial lesion in each mouse was shaved, and the probe tested on the fresh surface. The suction tube covering the probe enabled uniformly firm and reproducible contact with the tissue surface. Measurements were carried out in frequencies ranging from 1 to 6 GHz.

Each sampling took less than 1 s. The tested location was immediately dissected to prevent from mislocating the recorded region. When a recorded tissue was dissected, it was fixed in 10% formalin solution (24–48 h) and prepared for H&E staining, followed by pathological evaluations. The histopathological analysis was just conducted on the tissue interface with the sensing probe without further sectioning, so the external face of the tested specimen was chosen for histological examination. Due to this trend, the sampling locations remain registered in each analysis. The suction force kept the lesion stable during the sampling and marked during the dissection for pathological processing. We estimated that the mislocation between the exact measured site and the analyzed tissue sample was about 0 mm. The data of mouse samples from both normal and tumoral surfaces were collected and plotted to evaluate any probable calibration and meaningful difference between these two lesions.

### 2.3. GHz Response Classification in Breast Tissue Samples

After performing the standard protocols in the intraoperative frozen-section pathology and before transferring dissected tumor tissue to the formalin reservoir, measurements were taken by applying the suction to the tissue selected by the pathologist. Then the exact recorded tissues were dissected via punch biopsy and prepared for histopathological evaluation by pathologists. The central area of the specimen with a radius of 0.5 mm was the main area for histological analysis. This step obtained calibration data for benign, malignant, and fatty breast tissues.

In the next step, the probe was used for scanning a piece of tumor margin by multiple measurements from different places of the tumor margin. The location of each test was determined carefully. For correct coincidence between *S*_11_ and pathological data, the samples were flattened out after measurement in 10% formalin solution by putting a 200 gr weight on it for 10–15 min. This method prevents any tissue reshaping and helps to improve the quality of pathological evaluations and data analysis.

### 2.4. Statistical Analysis

Asymptotic significance or *p*-value of the Chi-square with a confidence interval of 95% was calculated to assess the significance of the differences between the *S*_11_ magnitude and the histopathological status of tumor margins, which determines the statistical significance of the relationship. Receiver Operating Characteristic curve (ROC) analysis was used to calculate the Area Under the Curve (AUC). These two kinds of statistical analysis were widely used by our and other groups to achieve meaningful correlated results between an electrical sensing approach and gold-standard biological results such as histopathology [29,30,31]. However, other statistical methods, such as ANOVA [32,33], could be applied as an accurate method to improve the results of statistical analyses, which will be used in the next trials of this research in future. A cut-off value for *S*_11_ magnitude as the GHz parameter was extracted in small samples. All statistical analyses were performed using commercially available software (IBM SPSS Statistics for Windows version 26).

### 2.5. Ethics

All research procedures and reporting were performed according to the Code of Ethics of the World Medical Association (Declaration of Helsinki), ethical principles, national norms, and standards for conducting Medical Research in Iran. All of the human tests were performed under the license of the Ethics Committee of Tehran University of Medical. Institutional review board (IRB) or research ethics committee (REC) approval is IR.TUMS.VCR.REC.1397.532. Patient information and data were kept confidential and secure at all times.

This study was eligible for a waiver of consent because it has no intervention in the pathological processes. No harm is posed to study participants and no diagnostics are interrupted. Absence of harm is defined as the probability and magnitude of damage or discomfort not greater than ordinarily encountered during the performance of routine pathopsychological tests, with no effect on the course of disease management.

## 3. Results

**Figure 2:**
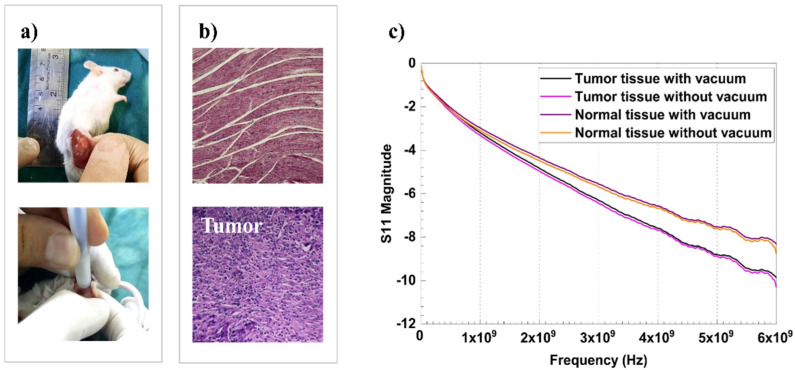
GHz measurement on a mouse model. (**a**) Tumor tissue and applying the GHz probe on it. (**b**) Normal mouse tissue with H&E assay representing the fibrotic connective tissue and cancerous mouse tissue with H&E assay representing a hypercellular region and high nucleus-to-cytoplasm size ratio. (**c**) Frequency-dependent behaviour of *S*_11_ parameter for normal and cancerous tissues with or without applying suction force.

Figure 2 presents the results of GHz spectroscopy on normal and tumor regions of the mouse model (Figure 2a) that had been tumorized by TNBC 4T1 cell injection. The results revealed a drastic difference between healthy and cancerous tissues, presenting −7 dB as the cut-off value for the differential diagnosis (Figure 2c). Normal mouse tissue presents fibrotic connective tissues, while cancerous mouse tissue presents a hypercellular region and high nucleus-to-cytoplasm size ratio in H&E assays (Figure 2b). No electrical shock occurred on the mouse during the analysis. Device measurements were non-destructive and had no destructive effect on the specimens for the histopathological procedure.

**Figure 3:**
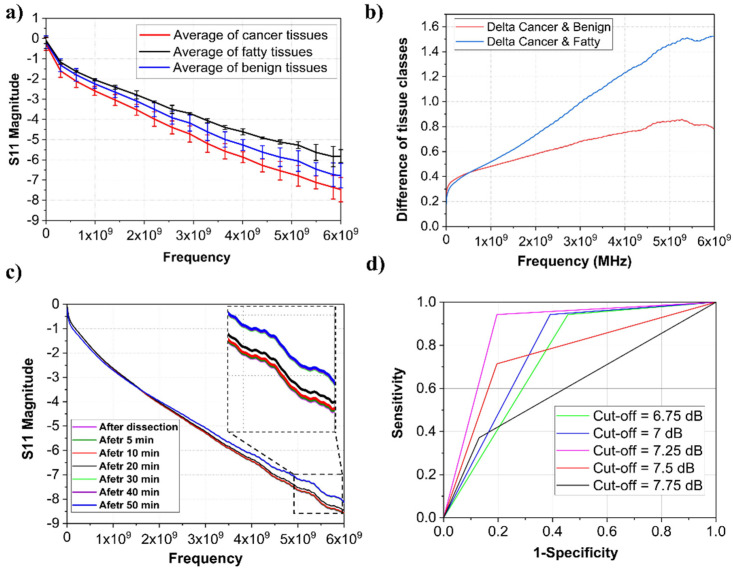
(**a**) mean values of *S*_11_ magnitude in three categories of fatty, benign, and malignant breast tissues. (**b**) Differences of each category versus tumor spectrum. (**c**) Variation of measured *S*_11_ by the time after dissection with magnification. (**d**) The ROC curve for different cut-offs.

Subsequently, the work was spread out to human breast cancer patients. Obtaining calibration data for fatty, normal, and malignant breast tissues in an ex vivo measurement setup was the first attempt in this step. GHz spectroscopy was conducted on 127 tiny margin samples selected by the pathologist from 19 recruited patients (16 invasive ductal and 3 invasive lobular carcinomas, 15 adjuvant and 4 neuadjuvant patients) after doing all standard procedures of frozen pathology (including touch imprint, sample preparation, tissue sectioning, Hematoxyline/Eosin Staining, etc.). Then, H&E assays were made from tested margins, and sent for microscopy and final histopathological diagnosis as the gold standard. Also, nine immunohistochemistry analyses (IHC) were performed in challenging samples. Two *S*_11_ magnitude measurements were carried out for each sample, and the mean value was reported in the manuscript data. A distinctive pattern was observed in the *S*_11_ magnitude diagram by averaging all data of three categories (fatty, benign, and malignant breast tissues) (Figure 3a). For a precise selection of the most sensitive frequency to extract the data, the differences of each category versus malignant breast tissue were calculated (Figure 3b). The difference in each couple categories (fatty vs. cancerous tissue, benign vs. cancerous tissue) obviously increases by increment in the frequency. The most significant difference occurred in about 5 to 6 GHz. Thus, the *S*_11_ magnitude in f = 6 GHz was selected to be extracted from each measurement set as the classification parameter. The mean of *S*_11_ magnitude in 6 GHz of all healthy breast tissues is about 20% higher than in malignant tissues.

Furthermore, measurement results were unchanged up to 30 min after tissue dissection (Figure 3c), and the validation may have been missed by decreasing the water content.

For finding the most precise cut-offs of *S*_11_ magnitude for tissue characterization, five different border values of −6.75 dB, −7 dB, −7.25 dB, −7.5 dB, and −7.75 dB were selected, and the ROC curve was calculated (Figure 3d). The most Area Under the Curve (AUC) equal to 0.87 belongs to −7.25 dB as the *S*_11_ magnitude cut-off value (Table 1).

PPV, NPV, accuracy, and AUC calculated for each cut-off are reported in Table 1. As illustrated in Table 1, the best sensitivity and specificity (94% and 83%) were achieved at −7.25 dB as the cut-off classification value with a *p*-value < 0.001.

**Figure 4:**
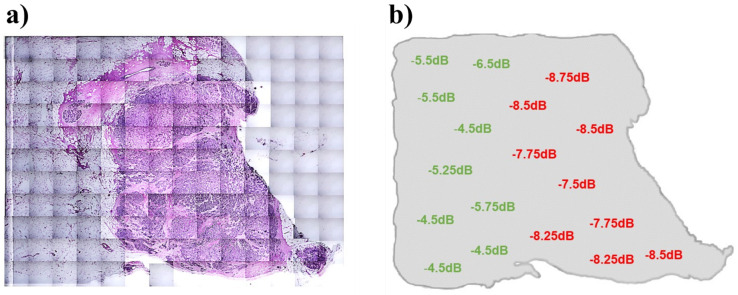
GHz spectroscopy on human tumor margins. (**a**) H&E assay of a measured margin sample, 2.7 × 2.7 cm. (**b**) *S*_11_ magnitude measurements on the selected margin in 18 points. Pathologically negative and positive points are represented in green and red, respectively. The obtained pattern is completely matched with the H&E assay.

In the last step of this work, margin evaluation by GHz spectroscopy was carried out. Measurements of 86 in vitro breast samples from 22 patients (15 invasive ductal and 7 invasive lobular carcinomas, 15 adjuvant and seven neoadjuvant cases) were conducted within the proposed method. After doing all standard procedures of frozen pathology, margin samples with average dimensions of 20 × 30 mm^2^ were selected by the pathologist for GHz spectroscopy (Figure 4a). Each tissue sample was scanned in a mesh-like pattern with the coaxial cable, and *S*_11_ magnitude measurement and recording took place in distance intervals of 5 mm. The average number of measures for each margin sample was about 15.

Then, H&E assays were made from tested margins, and sent for microscopy imaging and final histopathological diagnosis as the gold standard. Furthermore, 11 IHC analyses were performed in challenging samples.

The GHz probe was also applied in in vitro breast tumor margin evaluations. In this regard, mesh patterns were assumed for the margin with a feature size of 5 mm^2^. Hence, about 15 points were recorded by the probe in each margin. Recorded data resulted in diagnostic scoring based on our suggested calibration. Then, the H&E image of the margin was taken and evaluated by a pathologist. Matching the GHz probe scores and H&E diagnosis of mesh patterns (Figure 4a,b) showed more than 85% accuracy in margin scoring by the probe.

## 4. Discussion

GHz range EM waves have been demonstrated as effective detection elements in interaction with biological tissue surfaces due to the state and concentration of their electrolyte and water molecules [34].

**Figure 5:**
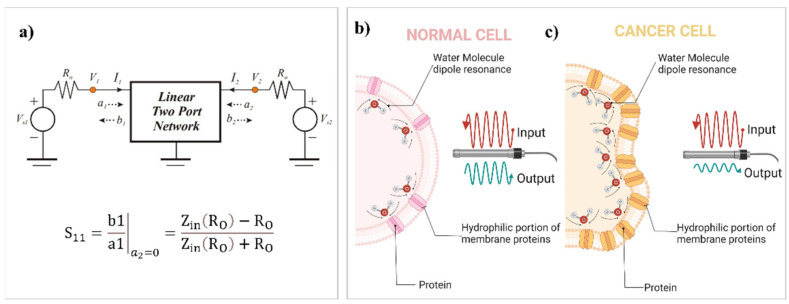
(**a**) Schematic view of an electrical network with two ports in a high-frequency region to describe scattering parameter, *S*_11_. (**b**) Schematic view of protein content and water distribution of a normal cell, which results in an increased *S*_11_ parameter in normal tissue due to less resonant membrane-bound waters associated with decreased expression of membrane proteins. (**c**) Schematic view of protein content and water distribution of a cancer cell, which results in a decreased *S*_11_ parameter in cancerous tissue due to more resonant membrane-bound waters associated with increased expression of membrane proteins.

Tumor tissues are reported to have significantly higher water and sodium content than normal tissues, which is the reason behind their variations with respect to dielectric properties [26,35]. Understanding tumor detection mechanism in GHz ranges requires careful analysis of their cellular structure, protein content, and water distribution differences from normal ones. Rapid growth and proliferation of cancer cells lead to overall increased expression of proteins (including membrane proteins), in contrast to normal cells [36]. It is also known that proteins acquire more surface charges in malignant tumors [24,37]. Proteins have both hydrophilic and hydrophobic components that may react to dipolar molecules [38]. Proteins expressed on the cell membrane surface attract more water molecules as a dominant component in the cellular medium to form “bound water” [24,37,39]. These dipoles can be oriented by an oscillating electric field in frequencies less than 20 GHz (the resonance frequency of the free water molecule is 25 GHz). Thus, bound water accounts for increased electromagnetic energy absorption and cell response in GHz frequencies lower than 20 GHz (e.g., 6 GHz) [37]. In other words, higher content of expressed membrane proteins and bound water in cancer cells may play a crucial role in distinct responses between normal and tumoral regions in the frequency range of our study [18,24,37,39] (Figure 5). In terms of electromagnetic wave dispersion language, wave parameters could be quantified to describe the interaction between wave and matter [40]. The presence of a higher amount of bound water molecules in cancer lesions may change the wave scattering parameters; it could be assumed as a diagnostic indicator to distinguish between normal and cancer tissue surfaces. Scattering parameters also define the input/output relationships between ports in an electrical system (Figure 5). In a two-port device (used in this research as a sensing element (Figure 1d)), if *a*_1_ wave was transmitted from port1, *a*_2_ was transmitted from port2, *b*_1_ was returned to port1, and *b*_2_ was returned to port2 (Figure 5a), we can define *S*_11_, the main sensing parameter, as:

$$S_{11}=\frac{b1}{a1}{|}_{a_{2}=0}=\frac{Z_{in}\left(R_{O}\right)-R_{O}}{Z_{in}\left(R_{O}\right)+R_{O}}$$

In which *Z_in_* is entrance impedance, and *R*_0_ is transmission line resistance. After recording *S*_11_ signals from, on average, 15 points of 86 normal and cancerous tissues, we observed that 83% of the recorded data were specifically in the range of proposed calibration for neoplastic, and normal lesions had been confirmed by histopathology (Table 1). Due to our recorded calibration, the *S*_11_ between −7.25 dB and −9.5 dB (in the frequency range of 6 GHz) are in correlation with the amount of bound water molecules found in neoplastic tissue surface while the *S*_11_ values between −4.5 dB and −7.25 dB are equal to the resonance response of water molecules of non-neoplastic tissue surfaces. This system has the ability to scan all over the tissue surface without any invasive measurement. Hence, it could be used both in vitro and in vivo for margin assesment if the sensitivity and specificity maintain their acceptable range after subsequent investigations and trials.

More studies on H&E assays of these samples revealed that some relationships may exist between hyalinized fibrotic breast tissues, reduced *S*_11_ magnitude parameters, and the intercoastal water content of the tissue (apart from bound intracellular water content) [41]. It is reported that fibrotic hyalinized breast tissue has a high water content compared to normal and fatty breast tissues [41]. This phenomenon may be a limitation in GHz-based spectroscopy of neoadjuvant (post-chemotherapeutic) breast tissue diagnosis because hyalinized benign tissues may show a response similar to high-risk lesions. However, in non-neoadjuvant cases, this spectorscopy could be a promising complementary diagnostic tool with fast scanning and the least invasive abilities.

## 5. Conclusions

In summary, a GHz probe with coaxial cable and suction force was applied for a distinction between normal and cancerous lesions in mouse model in vivo and human breast tumor margins in vitro. *S*_11_, the reflection dispersion parameter of each recorded specimen in frequencies ranging from 0.1 to 6 GHz was extracted and categorized by their histological analyses. We know that the tissues in the GHz range’s permittivity, conductivity, and dielectric properties are correlated with the *S*_11_ parameter.

The coaxial sensing probe has a diameter of 0.5 mm. With the assistance of suction force, such a small probe facilitated the desired contact between the sensor and the resected tissue surface. Thus, by attachment of the probe to the tissue surface, the response signal was recorded in less than 10 s. Using the experimentally achieved cut-off results showed that the *S*_11_ between −4.5 dB and −7.25 dB are the responses of normal tissue surfaces to incoming GHz wave while the *S*_11_ between −7.25 dB and −9.5 dB are for cancerous tissue surface of breast samples.

More than 60 breast samples were tested by the probe, among which 30 samples were malignant, and others were normal or benign lesions. GHz probe calibration scoring was correctly matched with 49 of the samples. We achieved a scanning pattern of the tissue surface with the assistance of the probe, which was rechecked by histopathological evaluation.

Due to calibrated results by histopathological gold standards, the GHz probe showed a sensitivity and specificity of 94.7% and 83.1% in evaluating breast tumor margins, respectively.

Furthermore, the flexibility of this real-time probe helps us to use it easily in the pathology room and during surgery without any requirement for a standing probe or complicated setup.

## Figures and Tables

**Table 1:** Defining a cut-off value for samples.

Cut-Off	AUC	*p*-Value	Sensitivity	Specificity	Accuracy
Cut-off = −6.75 dB	0.743	0.00002	%94.3	%54.3	%65.3
Cut-off = −7 dB	0.776	0.000001	%94.3	%60.8	%70
Cut-off = −7.25 dB	0.874	0.0000000001	%94.7	%83.1	%86.6
Cut-off = −7.5 dB	0.759	0.000006	%71.4	%80.4	%77.9
Cut-off = −7.75 dB	0.620	0.03	%37.1	%86.9	%73.2

## Data Availability

The data presented in this study are available on request from the corresponding author. The data are not publicly available due to their containing information that could compromise the privacy of research participants.
